# Functional *BCL-2* regulatory genetic variants contribute to susceptibility of esophageal squamous cell carcinoma

**DOI:** 10.1038/srep11833

**Published:** 2015-07-01

**Authors:** Wenting Pan, Jinyun Yang, Jinyu Wei, Hongwei Chen, Yunxia Ge, Jingfeng Zhang, Zhiqiong Wang, Changchun Zhou, Qipeng Yuan, Liqing Zhou, Ming Yang

**Affiliations:** 1 State Key Laboratory of Chemical Resource Engineering, Beijing Laboratory of Biomedical Materials, College of Life Science and Technology, Beijing University of Chemical Technology, Beijing, China; 2 Department of Gastrointestinal Surgery, Shandong Cancer Hospital, Shandong Academy of Medical Sciences, Jinan, Shandong Province, China; 3 Clinical Laboratory, Shandong Cancer Hospital, Shandong Academy of Medical Sciences, Jinan, Shandong Province, China; 4 Department of Radiation Oncology, Huaian No. 2 Hospital, Huaian, Jiangsu Province, China

## Abstract

B-cell lymphoma-2 (BCL-2) prevents apoptosis and its overexpression could promote cancer cell survival. Multiple functional *BCL-2* genetic polymorphisms, such as rs2279115, rs1801018 and rs1564483, have been identified previously and might be involved in cancer development through deregulating BCL-2 expression. Therefore, we examined associations between these three polymorphisms and esophageal squamous cell carcinoma (ESCC) susceptibility as well as its biological function *in vivo*. Genotypes were determined in two independent case-control sets consisted of 1588 ESCC patients and 1600 controls from two regions of China. *Odds ratios* (*ORs*) and 95% *confidence intervals* (*CIs*) were calculated by logistic regression. The impact of the rs2279115 polymorphism on *BCL-2* expression was detected using esophagus tissues. Our results demonstrated that the *BCL-2* rs2279115 AA genotype was significantly associated with decreased ESCC risk compared with the CC genotype (*OR* = 0.72, 95% *CI* = 0.57–0.90, *P* = 0.005), especially in nonsmokers (*OR* = 0.42, 95% *CI* = 0.29–0.59, *P* = 0.001) or nondrinkers (*OR* = 0.44, 95% *CI* = 0.32–0.62, *P* = 0.002). Genotype-phenotype correlation studies demonstrated that subjects with the rs2279115 CA and AA genotypes had a statistically significant decrease of *BCL-2* mRNA expression compared to the CC genotype in both normal and cancerous esophagus tissues. Our results indicate that the *BCL-2* rs2279115 polymorphism contributes to ESCC susceptibility in Chinese populations.

Apoptosis has been widely recognized as a well controlled and conserved process which is crucial for the normal development and function of multiple organisms^1^. Deregulations of apoptosis lead to either inappropriate killing of vital cells or survival of unwanted cells, which has been considered to be a hallmark of most cancers^2^,^3^. B-cell lymphoma-2 (BCL-2) family proteins are essential regulators of apoptosis and consists of both pro- and anti-apoptotic members, which all share sequence homology in their BCL-2 homology domains^4^. These proteins can promote cell survival (BCL-2 and BCL-xL), initiate cell killing (BIM, PUMA and BID) or activate the effecter pathways of apoptosis (BAX and BAK)^4^. *BCL-2* was firstly identified during the investigation of t(11;14) chromosome translocation in B-cell lymphoma^5^. BCL-2 protein locating on intracellular membranes prevents apoptosis in response to various death inducing stimuli and its overexpression could promote cancer cell survival^5^. The discovery of BCL-2 established a new paradigm in cancer biology, namely that apoptosis defects give cells selective survival superiority^5^.

As one of the most common and fatal malignancies worldwide, esophageal squamous cell carcinoma (ESCC) shows a relatively high incidence in Asian including China^6^. Cigarette smoking, heavy ethanol consumption, micronutrient deficiency as well as dietary carcinogen exposure have been identified as main environmental etiological factors of ESCC^7^,^8^. Accumulated evidences indicate that genetic makeup may also contribute to ESCC susceptibility. For example, ESCC genome-wide association studies (GWAS) highlight the involvement of single nucleotide polymorphisms (SNP) in cancer development, alone and in combination with environmental risk factors^9^,^10^,^11^,^12^,^13^,^14^.

In ESCC, BCL-2 plays its role in regulating cancer cell growth, especially in the early stage. Additionally, BCL-2 expression has been positively associated with cancer cell differentiation and inversely with disease progression^15^. There are multiple functional genetic polymorphisms have been identified in the *BCL-2* gene locus which is located on chromosome 18q21.3 and consists of three exons and two promoters. These two promoters show different functional properties. That is, *BCL-2* mRNA transcription is driven by the P1 promoter, while the P2 promoter acts as a negative regulatory element^16^,^17^. There is a functional rs2279115 (−938 C > A) promoter SNP in the inhibitory P2 promoter^18^. Interestingly, the rs2279115 A allele may render a better interaction with TP53, leading to a decrease in the BCL2 expression, an up-regulated programmed cell death or reduced longevity of transformed cells, and thus a subsequent decrease in the risk of malignances, such as squamous cell carcinoma of the head and neck (SCCHN)^19^. There is only one study with a relatively small sample size investigated the role of this SNP in the etiology of ESCC without genotype-phenotype association investigations^20^. Considering the importance of BCL-2 in tumorigenesis, we hypothesized that the *BCL-2* functional polymorphisms (rs2279115, rs1801018 and rs1564483) might be also involved in ESCC development through deregulating BCL-2 expression. To test this hypothesis, we conducted a two-stage case-control study of ESCC. To validate the biological function of *BCL-2* rs2279115 genetic variant *in vivo*, we detected the association between its genotypes and *BCL-2* mRNA expression levels in normal and cancerous esophagus tissues.

## Materials and Methods

### Study subjects

Two case-control sets were included in the current study. Huaian case-control set consists of 588 ESCC cases from Huaian No. 2 Hospital (Huaian, Jiangsu Province, China) and sex- and age-matched 600 healthy controls. Jinan case-control set contains 540 patients with ESCC from Shandong Cancer Hospital, Shandong Academy of Medical Sciences (Jinan, Shandong Province, China) and sex- and age-matched (±5 years) 550 controls. We used the group match considering sex- and age-match between cases and controls. The detailed information about the two case-control sets has been reported previously^21^,^22^,^23^. Twenty-nine ESCC tissues and twenty nine paired esophagus normal tissues adjacent to the tumors were obtained from surgically removed specimens of patients in Huaian No. 2 Hospital. The normal tissues sampled at least 2 cm away from the margin of the tumor. All subjects were ethnic Han Chinese. This study was approved by the Institutional Review Boards of Huaian No. 2 Hospital and Shandong Cancer Hospital, Shandong Academy of Medical Sciences. At recruitment, the written informed consent was obtained from each subject. The methods were carried out in accordance with the approved guidelines.

### Genotyping of BCL-2 polymorphisms

Three *BCL-2* candidate SNPs were analyzed by the MassArray system (Sequenom Inc., San Diego, California, USA). A 5% blind, random sample of study subjects was genotyped in duplicates and the reproducibility was 98.8%. To reduce the costs of the study, we genotyped the *BCL-2* rs2279115 SNP in the validation set using PCR-based restriction fragment length polymorphism (RFLP). The genotyping primers used for amplifying DNA segments with the SNP site were 5′-GCATTTGCTGTTCGGAGTTT-3′ and 5′-TTCGCAGAAGTCCTGTGATG-3′. The 25 μL PCR reaction mixture contains 0.2 mmol/L of deoxynucleoside triphosphate, 0.1 mmol/L of each primer, 100 ng of DNA, 1.0 U of rTaq DNA polymerase (TaKaRa), 1.5 mmol/L MgCl_2_, and 1 × reaction buffer. The PCR profile included an initial 2 minutes melting step at 95 °C, followed by 35 cycles of 30 seconds at 94 °C, 30 seconds at 60 °C, 30 seconds at 72 °C, and a final 10 minutes elongation step at 72 °C. Restriction enzyme *Bcc*I (New England Biolabs) was used to distinguish the rs2279115 C > A genotypes. A 15% random sample was reciprocally tested by different person, and the reproducibility was 99.0%.

### Real-time Analysis of BCL-2 mRNA

Total RNA was extracted from ESCC tissue samples using TRIzol Reagent (Invitrogen) and converted to cDNA using the ReverTra Ace qPCR RT Kit (TOYOBO). *BCL-2* mRNA expression in cancerous and normal esophagus tissues was examined using the SYBR-Green real-time quantity PCR (qPCR) method as described previously^23^,^24^,^25^. Gene expression for *BCL-2* and *β-actin* as an internal reference gene was carried out using the ABI 7500 real-time PCR system in triplicates. The primers used for *BCL-2* were 5′-TCGCCCTGTGGATGACTGA-3′ and 5′-CAGAGACAGCCAGGAGAAATCA-3′; and for *β-actin* were 5′-GGCGGCACCACCATGTACCCT-3′ and 5′-AGGGGCCGGACTCGTCATACT-3′. The samples size of each qPCR assay is 10 μL. Relative gene quantitation for *BCL-2* was calculated by -delta delta ct methods. To control quality of the qPCR data, we repeated the qPCR assays for some specific sample if the ct values of the triplicates for this sample showed variations more than 0.5 in very few cases. Paired *t*-test was used to calculate the differences between individuals with different genotypes.

### Statistics

The differences in demographic variables and genotype distributions of *BCL-2* SNPs between ESCC cases and controls were examined using Pearson’s χ^2^ test. Unconditional logistic regression model was utilized to estimate associations between *BCL-2* genotypes and ESCC risk by *odds ratio* (*OR*) and their 95% *confidence intervals* (*CIs*). During calculating associations between functional SNP candidates in *BCL-2* and ESCC risk in Huaian case-control set, we used the common genotypes of rs2279115 (CC), rs1801018 (AA) and rs1564483 (GG) as the reference genotype. All *ORs* were adjusted for age, sex, drinking and smoking status, where it was appropriate. A *P* value of less than 0.05 was used as the criterion of statistical significance, and all statistical tests were two-sided. All analyses were performed with SPSS software package (Version 16.0, SPSS Inc., Chicago, IL).

## Results

There were no statistically significant differences between cases and controls for both case-control sets in terms of median age and sex distribution (all *P* > 0.05), which indicated that the frequency matching of age and sex was adequate (Table 1). More smokers were observed among ESCC cases compared with controls in both case-control sets (Huaian set: 74.3% vs. 33.8%, *P* < 0.001; Jinan set: 75.2% vs. 39.6%, *P* < 0.001). Similarly, there were more alcohol drinkers among patients than among control subjects in these two sets (Huaian set: 56.8% vs. 40.3%, *P* < 0.001; Jinan set 55.3% vs. 40.1%, *P* < 0.001).

The genotype frequencies of *BCL-2* candidate SNPs (rs2279115 C > A, rs1801018 A > G and rs1564483 G > A) are summarized in Table 2. The allele frequencies for rs2279115 C, rs1801018 G and rs1564483 A were 0.362, 0.084, and 0.375 in ESCC cases and 0.410, 0.098, and 0.338 in control subjects in Huaian training case-control set. All observed genotype frequencies in either controls or cases conform to Hardy-Weinberg equilibrium. Distributions of the rs2279115, rs1801018 and rs1564483 genotypes were then compared among patients and controls. Frequencies of rs2279115 CC, CA, and AA genotypes among ESCC cases differed significantly from those among controls (χ^2^ = 8.68, *P* = 0.013, *df* = 2), with the frequency of AA homozygote being significantly lower among patients than among controls (13.7% vs. 15.0%). However, no statistically significant differences of rs1801018 and rs1564483 genotypes were observed between cases and control subjects (both *P* > 0.05) (Table 2). Therefore, we did no other analyses of these two polymorphisms in the next studies.

Associations between genotypes of *BCL-2* rs2279115 C > A SNP and ESCC risk were calculated using unconditional logistic regression analyses (Table 3). The *BCL-2* rs2279115 A allele was shown to be a protective allele. Individuals with the rs2279115 CA genotype had an *OR* of 0.66 (95% *CI* = 0.50–0.88, *P* = 0.004) for developing ESCC in Huaian Set, compared with individual having the rs2279115 CC genotype. However, the rs2279115 AA genotypes had a marginally decreased risk for ESCC compared with the rs2279115 CC genotype (*OR* = 0.85, 95% *CI* = 0.70–1.03, *P* = 0.095). In Jinan set, carriers of the rs2279115 CA or AA genotypes were significantly associated with decreased ESCC risk (*OR* = 0.62, 95% *CI* = 0.50–0.77, *P* = 0.002, or *OR* = 0.49, 95% *CI* = 0.36–0.66, *P* = 4.2 × 10^−4^) (Table 3). In the pooled analyses, we observed that only individuals with the rs2279115 AA genotype had a 0.72-fold decreased risk to develop ESCC compared to the CC genotype carriers (95% *CI* = 0.57–0.90, *P* = 0.005) (Table 3). All *ORs* were calculated with adjustments of sex, age, smoking and alcohol drinking status.

Associations between genotypes of *BCL-2* rs2279115 genetic variant and ESCC risk was further examined by stratifying for age, sex, smoking and alcohol drinking status using the pooled data of two Chinese case-control sets (Table 4). Compared with the *BCL-2* rs2279115 CC genotype, a significantly decreased risk of ESCC was associated with AA genotypes only among males (*OR* = 0.70, 95% *CI* = 0.54–0.91, *P* = 0.008), but not among females (*OR* = 0.74, 95% *CI* = 0.42–1.32, *P* = 0.309). However, the *BCL-2* rs2279115 CC genotype was not significantly associated with ESCC susceptibility in males or females (*OR* = 0.86, 95% *CI* = 0.71–1.04, *P* = 0.123, or *OR* = 0.97, 95% *CI* = 0.66–1.42, *P* = 0.868). In age-stratified analyses, either rs2279115 CA or AA genotype was significantly associated with decreased risk in subjects aged older than 57 years (*OR* = 0.76, 95% *CI* = 0.60–0.96, *P* = 0.021, or, *OR* = 0.56, 95% *CI* = 0.40–0.78, *P* = 0.001). However, among subjects aged 57 years or younger, neither rs2279115 CA nor AA genotype showed impacts on ESCC risk (*OR* = 0.56, 95% *CI* = 0.40–0.78, *P* = 0.001, or, *OR* = 0.92, 95% *CI* = 0.65–1.29, *P* = 0.619).

Because tobacco smoking and alcohol drinking are both risk factors for ESCC, we then examined whether the *BCL-2* rs2279115 genetic variant influence ESCC susceptibility in combination with these pathogenic factors (Table 4). In nonsmokers, compared with the rs2279115 CC carriers, individuals with CA or AA genotype had a 0.70-fold or 0.42-fold decreased risk to develop ESCC (95% *CI* = 0.54–0.92, *P* = 0.010, or 95% *CI* = 0.29–0.59, *P* = 0.001). There was no significantly decreased risk for smokers with CA or AA genotype compared with CC smokers (both *P* > 0.05). Nondrinkers carrying rs2279115 CA or AA genotype showed significantly decreased risk to develop ESCC compared with CC carriers who did not drink (*OR* = 0.78, 95% *CI* = 0.61–0.99, *P* = 0.041, or *OR* = 0.44, 95% *CI* = 0.32–0.62, *P* = 0.002). However, there were no association between rs2279115 CA and AA genotypes and ESCC risk in drinkers (both *P* > 0.05) (Table 4).

Due to rs2279115 C-to-A change could influence *BCL-2* P2 promoter activity and gene expression in cancer cells, we investigated whether there is an allele-specific effect of rs2279115 SNP on *BCL-2* expression in esophagus tissues. We found that there were significantly lower *BCL-2* mRNA levels (mean ± SE) among carriers of the rs2279115 CA and AA genotypes compared to carriers of the CC genotype in normal esophagus tissues (0.083 ± 0.012 [n = 17] vs. 0.109 ± 0.021 [n = 12], *P* = 0.031) (Table 5). Similar results have also been observed in ESCC tissues (the rs2279115 CA and AA genotypes: 0.153 ± 0.022 [n = 17] vs. the CC genotype: 0.184 ± 0.045 [n = 12], *P* = 0.040) (Table 5).

## Discussion

In the current study, we investigated the association between three *BCL-2* functional candidate SNPs and ESCC susceptibility through a case-control approach. We found that only *BCL-2* rs2279115 polymorphism is significantly associated with decreased ESCC susceptibility in Chinese populations, with the rs2279115 AA genotype as the protective genotype. Genotype-phenotype correlation studies demonstrated that subjects with the rs2279115 CA and AA genotypes had a statistically significant decrease of *BCL-2* mRNA expression compared to the CC genotype in both normal and cancerous esophagus tissues. Our data support the hypothesis that SNPs in gene expression regulatory elements of tumor suppressor genes or oncogenes might impact genetic susceptibility of cancers.

The *BCL-2* rs2279115 polymorphism has been extensively studied in multiple cancer types, including ESCC. Liu *et al.* reported in a case-control study conducted in western China including 205 esophageal cancer patients and 224 controls^20^. They found that the *BCL-2* rs2279115 AA genotype was significantly associated with increased risk of developing esophageal cancer. In contrast, we found that subjects with the rs2279115 AA genotype have significantly decreased risk to develop ESCC in both Northern and Southern Chinese populations. There are several possible explanations for the insistent results. Firstly, Liu *et al.* only recruited 205 esophageal cancer patients to evaluate association between rs2279115 and esophageal cancer risk. The relative large sample size of the present study (1588 ESCC patients and 1600 controls) may provide more statistic power to the moderate effect of this genetic polymorphism on ESCC susceptibility. Secondly, we only included ESCC, but not esophageal adenocarcinoma in this study. Considering the distinct etiology and clinical behaviors of these two subtypes of esophageal cancer, we believe that mixed study subjects might also lead to the discrepancy.

Our results are consistent to functional relevance of rs2279115 polymorphism in SCCHN^19^. That is, the rs2279115 AA genotype may result in decreased BCL-2 expression, elevated apoptosis rates of cancer cells, and thus decreased risk of malignances^19^. Song *et al.* recently reported that hierarchical clustering analyses of whole-genome sequencing in 17 ESCC cases and whole-exome sequencing in 71 cases indicate that ESCC and HNSCC mutation spectra were intermingled, whereas esophageal adenocarcinomas were clearly distinctive from ESCC^26^. Therefore, it is biologically plausible that the functional *BCL-2* rs2279115 polymorphism influence ESCC genetics thoroughly through regulating BCL-2 expression and apoptosis *in vivo*.

The *BCL-2* rs2279115 polymorphism showed a consistent association with ESCC risk in two independent case-control cohorts. Additionally, our results are unlikely to be attributable to unknown confounding factors due to having relatively large sample sizes, significantly increased odd ratios with small *P* values. More importantly, our results on the genotype-phenotype relationship between the rs2279115 polymorphism and gene expression supports our conclusion. However, there might be several limitations in the current case-control study. For instance, since all ESCC cases were recruited from the hospital, inherent selection bias may exist. Therefore, the findings of our study warrant to be validated in a population-based prospective study in the future. In addition, relatively small sample size for non-smokers and non-drinkers should be further analyzed in a larger population.

In conclusion, we demonstrated that functional *BCL-2* rs2279115 SNP was associated with a significantly decreased risk of ESCC in Chinese populations, especially in nonsmokers or nondrinkers. Our data may support the hypothesis that genetic variants can influence gene regulation might be important modifiers of ESCC susceptibility. These results may lead to better understanding of ESCC etiology in different populations.

## Additional Information

**How to cite this article**: Pan, W. *et al.* Functional *BCL-2* regulatory genetic variants contribute to susceptibility of esophageal squamous cell carcinoma. *Sci. Rep.* **5**, 11833; doi: 10.1038/srep11833 (2015).

## Figures and Tables

**Table 1:** Distribution of selected characteristics among patients with esophageal squamous cell carcinoma and controls.

Variable	Huaian set			Jinan set		
	Cases	Controls	*P*-value^1^	Cases	Controls	*P*-value^1^
	No. (%)	No. (%)		No. (%)	No. (%)	
	588	600		1000	1000	
Sex			0.678			0.426
Male	413(70.2)	428(71.3)		776(77.6)	761(76.1)	
Female	175(29.8)	172(28.7)		224(22.4)	239(23.9)	
Age (year)^2^			0.725			0.474
≤59(or 56)	288(49.0)	300(50.0)		516(51.6)	500(50.0)	
>59(or 56)	300(51.0)	300(50.0)		484(48.4)	500(50.0)	
Smoking status			<0.001			<0.001
No	151(25.7)	397(66.2)		248(24.8)	604(60.4)	
Yes	437(74.3)	203(33.8)		752(75.2)	396(39.6)	
Drinking status			<0.001			<0.001
No	254(43.2)	358 (59.7)		447(44.7)	599(59.9)	
Yes	334(56.8)	242(40.3)		553(55.3)	401(40.1)	

^1^Two-sided χ^2^ test.

^2^Median ages of patients for Huaian set and Jinan set are 59 and 56 years.

**Table 2:** Associations between functional SNP candidates in *BCL-2* and ESCC risk in Huaian case-control set (Training set).

#	Identity	Location	Position^1^	Case	Common genotype No. (%)	Heterozygous genotype No. (%)	Rare genotype No. (%)	Common genotype	*OR*^2^ (95% *CI*) for heterozygote	*P*	*OR*^2^ (95% *CI*) for rare genotype	*P*
1	rs2279115	5' promoter	63319604	ESCC	242(41.2)	265(45.1)	80(13.7)	Reference	0.66(0.50–0.88)	0.004	0.85(0.70–1.03)	0.095
	(C > A)			Control	198(33.0)	312(52.0)	90(15.0)					
2	rs1801018	Exon 2	63318646	ESCC	493(83.8)	91(15.5)	4(0.7)	Reference	1.01(0.72–1.41)	0.972	0.77(0.39–1.52)	0.451
	(A > G)			Control	487(81.2)	108(18.0)	5(0.8)					
3	rs1564483	3’-UTR	63127421	ESCC	229(38.9)	278(47.2)	82(13.9)	Reference	1.30(0.98–1.70)	0.062	1.21(0.98–1.47)	0.068
	(G > A)			Control	271(45.1)	253(42.2)	76(12.7)					

Abbreviations: ESCC, esophageal squamous cell carcinoma; *OR, odds ratio; CI, confidence interval*; 3'-UTR, 3'-untranslated region.

^1^Position in NCBI build 38.

^2^Data were calculated by unconditional logistic regression, adjusted for sex, age, drinking and smoking status.

**Table 3:** Genotype frequencies of *BCL-2* rs2279115 genetic variant among patients and controls and their association with ESCC risk.

Genotypes		*BCL-2* rs2279115 C > A			
		Patients No. (%)	Controls No. (%)	*OR*^1^ (95% *CI*)	*P*-value
Huaian set		*n* = 587	*n* = 600		
	CC	242(41.2)	198(33.0)	Reference	
	CA	265(45.1)	312(52.0)	0.66(0.50–0.88)	0.004
	AA	80(13.7)	90(15.0)	0.85(0.70–1.03)	0.095
Jinan set		*n* = 1000	*n* = 1000		
	CC	416(41.6)	312(31.2)	Reference	
	CA	453(45.3)	516(51.6)	0.62(0.50–0.77)	0.002
	AA	131(13.1)	172(17.2)	0.49(0.36–0.66)	4.2 × 10^−4^
Total		*n* = 1587	*n* = 1600		
	CC	658(41.5)	510(31.9)	Reference	
	CA	718(45.2)	828(51.7)	0.86(0.73–1.02)	0.079
	AA	211(13.3)	262(16.4)	0.72(0.57–0.90)	0.005

Abbreviations: ESCC, esophageal squamous cell carcinoma; *OR, odds ratio; CI, confidence interval.*

^1^Data were calculated by logistic regression with adjustment for age, sex, smoking and drinking status.

**Table 4:** Risk of ESCC associated with*BCL-2* rs2279115 C > A genotypes by age, sex, smoking status and drinking status.

	*BCL-2* rs2279115 C>A				*BCL-2* rs2279115 C>A			
Variable	CC^1^	CA^1^	*OR*^2^ (95% *CI*)	*P*	CC^1^	AA^1^	*OR*^2^ (95% *CI*)	*P*
Sex								
Male	485/380	530/617	0.86(0.71–1.04)	0.123	485/380	159/207	0.70(0.54–0.91)	0.008
Female	173/130	188/211	0.97(0.66–1.42)	0.868	173/130	52/55	0.74(0.42–1.32)	0.309
Age (year)								
≤57	327/273	364/412	1.02(0.81–1.30)	0.843	327/273	96/131	0.92(0.65–1.29)	0.619
>57	331/237	354/416	0.76(0.60–0.96)	0.021	331/237	115/131	0.56(0.40–0.78)	0.001
Smoking status								
No	414/202	341/334	0.70(0.54–0.92)	0.010	414/202	108/109	0.42(0.29–0.59)	0.001
Yes	352/308	377/494	1.03(0.83–1.28)	0.808	352/308	103/153	1.01(0.81–1.50)	0.532
Drinking status								
No	306/202	341/334	0.78(0.61–0.99)	0.041	306/202	108/109	0.44(0.32–0.62)	0.002
Yes	352/308	377/494	0.98(0.78–1.23)	0.855	352/308	103/153	1.12(0.80–1.56)	0.514

Abbreviations: ESCC, esophageal squamous cell carcinoma; *OR, odds ratio; CI, confidence interval.*

^1^Number of case patients with genotype/number of control subjects with genotype.

^2^Data were calculated by logistic regression, adjusted for sex, age, smoking, and drinking status, where it was appropriate.

**Table 5:** An allele-specific effect of rs2279115 genetic variant on *BCL-2* mRNA expression in esophagus tissues.

*BCL-2* rs2279115 C>A			
	CC (mean ± SE)	CA and AA (mean ± SE)	
#	*n* = 12	*n* = 17	*P*-value
Normal esophagus tissues	0.109 ± 0.021	0.083 ± 0.012	0.031
ESCC tissues	0.184 ± 0.045	0.153 ± 0.022	0.040

Abbreviations: ESCC, esophageal squamous cell carcinoma.
