# Erratum: Functional *BCL-2* regulatory genetic variants contribute to susceptibility of esophageal squamous cell carcinoma

**DOI:** 10.1038/srep14020

**Published:** 2015-09-18

**Authors:** Wenting Pan, Jinyun Yang, Jinyu Wei, Hongwei Chen, Yunxia Ge, Jingfeng Zhang, Zhiqiong Wang, Changchun Zhou, Qipeng Yuan, Liqing Zhou, Ming Yang

Scientific Reports 5: Article number: 1183310.1038/srep11833; published online: 07012015; updated: 09182015

This Article contains an error in the affiliation of Jinyun Yang. The correct affiliation is listed below:

Department of Gastrointestinal Surgery, Huaian No. 2 Hospital, Huaian, Jiangsu Province, China

